# Case Report: Disease progression of renal cell carcinoma containing a novel putative pathogenic *KAT6A::NRG1* fusion on Ipilimumab- Nivolumab immunotherapy. A case study and review of the literature

**DOI:** 10.3389/fonc.2023.1111706

**Published:** 2023-02-02

**Authors:** Almas Dawood, Suzanne MacMahon, My-Anh Tran Dang, Maxine G. B. Tran, Axel Bex, Ekaterini Boleti, Soha El Sheikh

**Affiliations:** ^1^ Department of Cellular Pathology, Royal Free London Foundation Trust, London, United Kingdom; ^2^ The Centre for Molecular Pathology, The Institute of Cancer Research and The Royal Marsden NHS Foundation Trust, London, United Kingdom; ^3^ Specialist Centre for Kidney Cancer, Royal Free Hospital, London, United Kingdom; ^4^ Division of Surgery and Interventional Science, University College London, London, United Kingdom; ^5^ Research Department of Pathology, University College London (UCL) Cancer Institute, London, United Kingdom

**Keywords:** renal cell carcinoma, Nrg1, KAT6A, fusion, mutation, immunotherapy

## Abstract

Renal cell carcinoma still carries a poor prognosis despite therapeutic advancements. Detection of genetic mutations is vital in improving our understanding of this disease as well as potential role in targeted therapy. Here we present a case of a 49 year old man with an aggressive renal cell carcinoma bearing a novel pathogenic *KAT6A::NRG1* fusion. We will explore the clinical presentation, histological and molecular diagnostics, treatment and disease progression. We will discuss the relevance of this unique fusion and comparisons with cancer cases with similar genetic mutations. Further research is warranted for such cases, in order to facilitate better targeted treatments.

## Background

Advancements in the detection of genetic mutations offer a better understanding and classification of renal tumours, as well as provide the potential for specific targeted therapies. Neuregulin 1 (NRG1) and KAT6A are both oncogenes, and their mutations are rare, but have been reported in a range of tumours, including as components of gene fusions. Chromosomal rearrangements involving the NRG1 gene, which lead to a functional chimeric protein, have been found to act as independent oncogenic drivers in 0.2% of solid tumours overall, with a wide variety of fusion combinations across cancer types, rising to roughly 25% in the rare invasive mucinous adenocarcinoma of the lung (1). In renal cell carcinoma, the incidence of NRG1 mutations in mass molecular screening studies is around 0.5% in 2 large studies (1, 2). The biology of NRG1 fusions is exceedingly complex, heterogeneous, and difficult to study because of their rarity. NRG1, located on chromosome 8 (8p12 region) (3), encodes many isoforms and proteolytically cleaved forms; secreted, membrane-bound, cytoplasmic, and nuclear. Isoforms containing the transmembrane domain, once cleaved, can act as ligand for the tyrosine kinase receptors for the epidermal growth factor receptor (ERBB) family.

Here we present a case of a renal tumour with a unique and previously unreported *KAT6A::NRG1* fusion, and provide valuable insight into histopathological and molecular findings instead of NRG1 fusion RCCs, which had always been previously detected in mass retrospective molecular profiling histology-agnostic studies of large solid tumour cohorts.

## Case presentation

A 49-year-old man presented with mildly raised creatinine (125µmol/L) and mild proteinuria. A CT scan of the abdomen and pelvis showed an 8.7 x 6.4cm left renal tumour with clinically suspicious enlarged para-aortic lymph nodes.

**Figure 1:**
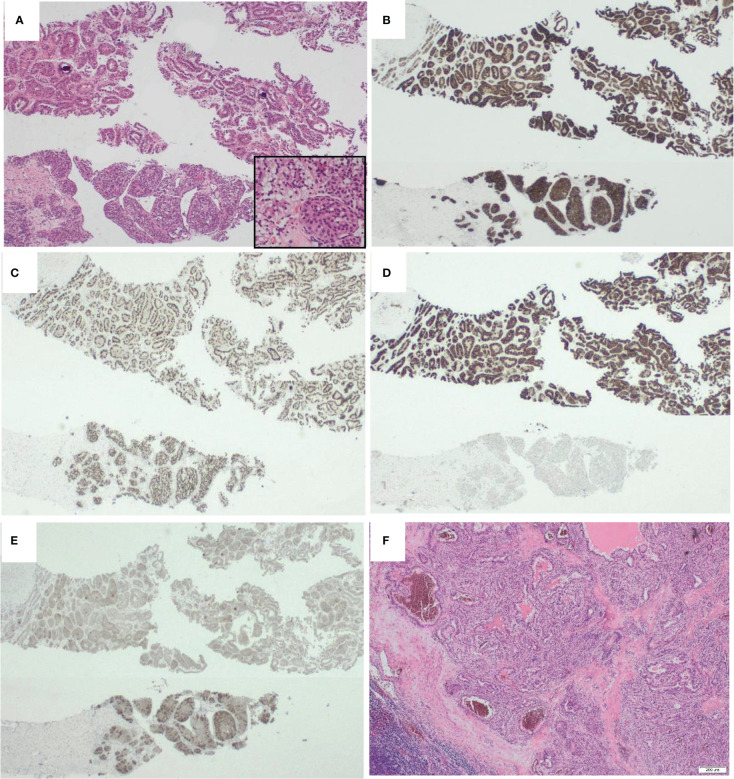
Morphological appearances of KAT6A::NRG1 fusion Renal cell carcinoma. **(A)** On H&E, the tumour had heterogenous morphology with areas had tubular architecture and resembled type 2 papillary renal cell carcinoma (top) while others had papillary areas with subnuclear vacuolation and more solid nests (inset). B-E are represetative of the immunohistochemical profile. Whereas some markers such as RACEMASE **(B)** and PAX8 **(C)** were diffusely expressed in all areas (cytoplasmic and nuclear resectively), CK7 **(D)** was almost exclusively expressed in the tubular areas while Melan A **(E )** was more strongly expressed in the solid areas. Interestingly, the morphology of the lymph node metastatic deposits **(F)** resembled clear cell papillary renal cell carcinoma. {Magnification x200 **(A)** and x100 **(B-F)**.

A core biopsy of the renal mass was taken, and showed renal parenchyma infiltrated by a neoplasm of variable patterns of growth and morphology. There were areas of tumour composed of solid nests and islands of epithelioid cytoplasm. These showed intracytoplasmic vacuoles. In other places, the tumour showed focal papillary formation lined by oncocytic cells. Nuclear grade was 2. There was no sarcomatous change or necrosis. (**Figure 1**)

Immunohistochemistry showed the tumour cells were positive for PAX-8 confirming renal origin. CD10, vimentin, SDHB were also positive. In addition, there was heterogenous expression of CK7, AMACR and Melan A (**Figure 1**). Tumour cells were negative for CK20, CAIX, GATA-3 and CD117. The tumour thus had mixed architectural and cytological features that overlapped with but did not completely fit with commoner types of renal cell carcinoma, together with the co-expression of epithelial and melanocytic markers. The preliminary diagnosis was of a probable MiT translocation renal cell carcinoma since histologically, the most typical cases show a mixed papillary and nested pattern with a mixture of cells with clear and granular/oncocytic cytoplasm, and an identical immunohistochemical profile (4).

The patient proceeded to radical nephrectomy and resection of the para-aortic lymph nodes.

Pathological examination of the radical nephrectomy showed an 82mm tumour, with a variegated golden yellow and tan-brown cut surface and no obvious necrotic areas. Histologically, the tumour showed similar features to the core biopsy, including the variable morphological patterns [tubular and solid nested areas] and clear to granular eosinophilic cytoplasm. There were areas reminiscent of clear cell papillary renal cell carcinoma and type 2 papillary renal cell carcinoma. The immunohistochemical profile was identical to the core biopsy results (**Figure 1**). The tumour invaded the renal sinus and perinephric fat and four out of five para-aortic lymph nodes submitted were involved by metastatic carcinoma. The lymph node metastasis resembled the morphological appearance of clear cell papillary renal cell carcinoma (**Figure 1F**). This suggests dissociation between the morphological appearances of this tumour and its behaviour, or the usual indolent behaviour of clear cell papillary carcinomas. The TNM (8^th^ edition) stage was pT3a N1 R0.

TFE3 Interphase fluorescent *in-situ* hybridisation (FISH) performed on formalin-fixed paraffin embedded tissue sections, using dual-colour break-apart probes was negative.

## Molecular findings

Molecular testing was conducted on the excised tumour. DNA-based next generation sequencing (NGS) confirmed the lack of pathogenic variants in pre-determined renal cancer-related genes: VHL, FH, SDHA, SDHB, SDHC, SDHD, TCEB-1, TSC1, TFE3, TSC2, MET, MTOR and BRAF.

**Figure 2:**
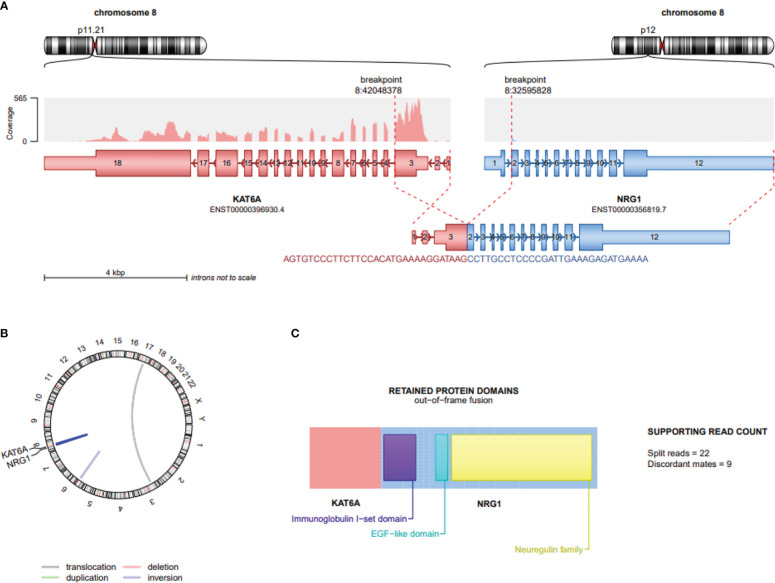
Schematic depiction of gene fusion involving KAT6A and NRG1 in Renal cell carcinoma. **(A)** Location of KAT6A and NRG1 on chromosome 8; the distance between these 2 loci is approximately 9,155kb. Exon 3 of KAT6A (NM_032427.4/ENST00000396930.4) is disrupted, inverted and fused to the 5’ end of exon 2 of NRG1 (NM_016215.4/ENST00000356819.7) depicting the 61 bases lying on the breakpoint **(B)** Visualization of whole genome using Circos plot highlighting deletions (red) and duplications (green), while connections within the circle indicate fusion transcripts. An interchromosomal translocation is marked by grey line, while intrachromosomal translocations are blue; KAT6A::NRG1 dark blue and an inversion pale blue. **(C)** The predicted protein structure indicates intact functional domains including exon 2 containing Ig–like C2 type domain and exon 6 containing the cardinal EGF-like domain. The Neuregulin structure codes for the c-terminal transmembrane domain and the cytoplasmic tail. This Figure was created by using the script draw_fusions.R with the STAR-alignment and Arriba fusion caller (https://github.com/suhrig/arriba).

RNA NGS analysis revealed a *KAT6A::NRG1* fusion, where exon 3 of KAT6A (NM_032427.4/ENST00000396930.4) is disrupted and inverted and subsequently fused to exon 2 of *NRG1* (NM_016215.4/ENST00000356819.7) (**Figure 2**).

Notably, and similar to all oncogenic NRG1 fusions, the NRG1 fragment is predicted to have a protein-coding potential and receptor-binding capacity with retained EGF-like domain and Ig-like domain. The exact role of the protein domains in the non-NRG1 partner gene remains to be elucidated.

## Clinical follow up and response to therapy

CT and MRI scans performed 3 months after the nephrectomy showed a lobulated soft tissue mass lesion of heterogeneous minimally increased T2 signal, and of high signal on STIR imaging. This demonstrated restricted diffusion and heterogeneous enhancement and was considered to represent a recurrent 2.5cm para-aortic lymph node at the level of the renal vein. Clinically, due to the short disease-free interval indicating rapid disease progression, a multidisciplinary recommendation was made to start with systemic therapy instead of local resection.

**Figure 3:**
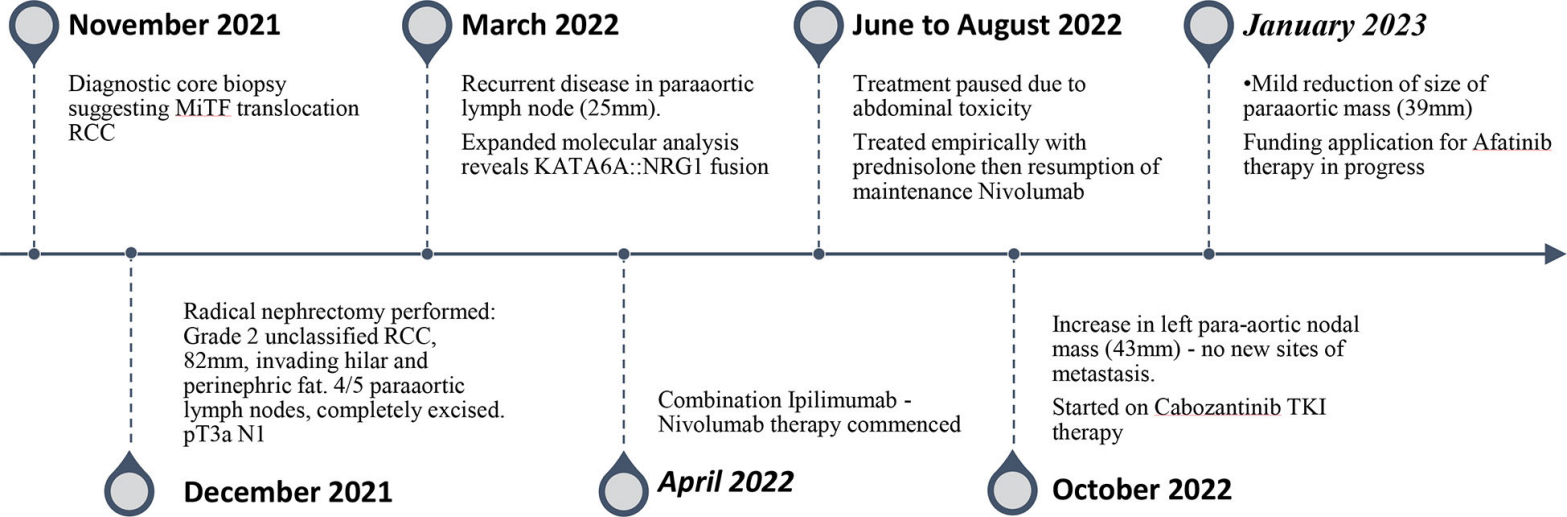
Diagnosis, follow up and treatment timeline of KAT6A::NRG1 RCC.

Based on the current UK treatment algorithms (5), non-clear cell RCC morphology and risk stratification, oncological management for the patient was commenced using first line combination immunotherapy with Ipilimumab (an anti-cytotoxic T-lymphocyte activation-4 (CTLA-4) agent) and Nivolumab (an anti-programmed death (PD)-1 agent) (6), with a plan for completion surgery if there was response to treatment. (Please see timeline in **Figure 3**).

A repeat CT Abdomen pelvis taken after 4 months of treatment with Ipilimumab-Nivolumab showed enlargement of the para-aortic lymph node to 3.6cm, increasing to 4.3cm on MRI at 6 months. This 72% increase in size from 2.5cm to 4.3 cm indicates disease progression despite on combination IPI-NIVO therapy. The patient was thus switched to Cabozantinib, a multi-targeted TKI that targets a range of receptor kinases including AXL, MET, and vascular endothelial growth factor (VEGF) receptors (7) that has shown efficacy after prior immunotherapy (8). Assessment of the clinical benefit from Cabozantinib after 3 months of treatment showed mild reduction in the size of the paraaortic mass to 3.9 cm. In addition, there are ongoing efforts to seek alternative funding for targeted NRG1-fusion therapies (**Figure 3**).

## Discussion

Renal cell carcinoma (RCC) is the seventh most common neoplasm in the developed world, constituting 2.2% of all cancer diagnoses new cases worldwide (9). There are 13 300 new cases of renal cancer every year in the UK (10), and over 400 000 worldwide (11). Despite significant improvements in the therapy of patients with advanced RCC, including novel therapies targeting key oncogenic pathways and immunotherapy, the prognosis of patients with metastatic renal cell carcinoma remains poor, and fewer than 10% of patients are alive five years after diagnosis (9) The majority of cases present between 60 and 65 years (12).

(3)Neuregulin-1 (NRG1) is the main physiological ligand to HER3 inducing dimerization of ErbB2/Her2 and ErbB3/HER3 receptors, resulting in their constitutive activation (1, 3) with subsequent modulation of downstream signalling pathways phosphoinositide 3-kinase-protein kinase B (PI3K-AKT), mitogen-activated protein kinase (MAPK), leading to growth, proliferation, decreased apoptosis, cellular migration and angiogenesis (13).

Other splice variants that do not encode transmembrane sequences are found either in the cytoplasm or, if they encode a nuclear localisation sequence, translocate to the nucleus and alter gene expression and this translocation is mediated by sequences around the Ig-like domain (14).

Here, we present an example of a previously unreported pathogenic *KAT6A::NRG1* fusion RCC. This novel *KAT6A::NRG1* gene fusion is biologically significant, because the predicted protein product has intact NRG1 functional domains, and because similar to previous studies that describe NRG1 fusions in other tumour types, they tend to be mutually exclusive with other known molecular drivers of that tumour (15).

Previously in RCC, three NRG1 fusion partners have been described: *PCM1-NRG1* (16), *RBPMS-NRG1* (1) and *NRG1-TNC* (17). In terms of tumour morphology, only the latter had been described, as an oncocytic papillary carcinoma with small nucleoli, not otherwise specified, sporadically showing cytoplasmic vacuolisation (17). These appearances partly resemble focal areas identified in our case, but the full spectrum of histomorphology and immunoprofile were lacking in that study, so it is not possible to pinpoint distinct common features.

The 5’ end fusion partner of NRG1 in our case is the oncogene KAT6A (also known as MOZ or MYST3) (18), a histone acetyltransferase with the MYST domain directly involved in protein proliferation, differentiation, and apoptosis. KAT6A was previously identified as a fusion partner with CREB binding protein in acute myeloid leukaemia (19). Solid tumours show recurrent amplification of the KAT6A gene including breast cancer, ovarian cancer, lung and colonic adenocarcinoma, and selective inhibitors have been discovered in studies (WM-8014 and WM-1119), at research stage (20).

Only a single report of KAT6A gene fusion in solid tumours has previously been published and this is the KAT6A-TFE3 renal cell carcinoma (21). In our case, exons 1-3 of KAT6A which encode the C terminal methionine-rich region were fused to NRG1. These form part of the serine methionine domain of the MYST protein family and are thought to possess potent transcriptional activation potential.

Our case overlapped morphologically with papillary and clear cell papillary renal cell carcinoma and these appearances were unrelated to biological behaviour. On immunohistochemistry with MiTF translocation carcinoma including the expression of Melan A, while Melan A expression can be attributed biologically in this group of tumours to activation of downstream targets of TFE3 including melanotic markers, we are not aware of the molecular basis of Melan A expression in our case, or its significance. While it may be an incidental finding, it does point to the fact that *KAT6A::NRG1* fusion RCC may be considered as a differential in histomorphologically unusual, TFE3 FISH negative, Melan A expressing RCC, together with other RCCs containing the subtle intrachromosomal *NONO-TFE3* (22) and *RBM10-TFE3* fusions (23) or those with TFEB translocation/amplification RCC.

### NRG1 fusions are clinically relevant and targetable

Our *KAT6A::NRG1* RCC case presented with lymph node metastasis and showed disease progression under Ipilimumab-Nivolumab-combination therapy. Assessment of the effectiveness of Cabozantinib is pending. A recent study has shown that NRG1-fusion non-small cell lung cancers are only modestly responsive to cytotoxic therapy, immunotherapy and targeted therapies [NO_PRINTED_FORM].

Early results of using HER-family kinase inhibitors in tumours with NRG1 fusions, for example afatinib in tumours with NRG1 fusions showed a substantial and maintained treatment response (13). In another trial, a patient (30, female) with poorly differentiated, metastatic KRAS wild-type pancreatic cancer also exhibited the *ATP1B1–NRG1* fusion. She initially showed a reduction in size of her liver metastasis when treated with afatinib. However, the dose had to be intermittently reduced due to side effects. A subsequent CT scan after three months showed progression of the lesion (15). However, these early results highlight a promising target for treatment. There is also the potential for greater benefit by specifically inhibiting HER3 (13).

One study (NCT04750824) (21) is evaluating the efficacy of afatinib following immunotherapy in patients with NRG1 fusion metastatic squamous cell lung cancer who received at least 1 line of systemic therapy. Results of a feasibility assessment by Gajra et al. (24) of the subgroup of patients with NSCLC showed that disease progression occurred following treatment with afatinib in 5 of the 26 patients (19.0%) who had received afatinib in the first-line through third-line therapeutic settings. Among the 23 patients who received a treatment other than afatinib in the first- through third-line settings, 5 patients (21.7%) experienced disease progression (following first-line therapy, 3 patients; following second-line therapy, 2 patients) (25).

Seribantumab is a monoclonal ErbB3 (Her3) antibody that inhibits ErbB3 activation, resulting in possible inhibition of ErbB3-dependent PI3K/Akt signalling (26). The FDA granted fast-track designation to seribantumab in 2016 for its development as a therapy in patients with NRG1–positive, locally advanced or metastatic NSCLC whose disease has progressed following immunotherapy. This designation was based on the results of the SHERLOC trial (NCT02387216) (27).

Furthermore, an ongoing trial currently recruiting is using MCLA-128, which is a HER2/HER3 bispecific antibody that blocks both NRG1 binding and HER2/3 dimerization, in locally advanced or metastatic NRG1 fusion cancers that have failed under prior standard therapy (28).

## Conclusion

This case demonstrated a renal tumour with a unique gene fusion of two different components both of which are oncogenic and associated with cancer in other organ systems. It adds to the pathologic and genetic spectrum of NRG1 fusion carcinomas. The tumour manifested an unusual hybrid morphological pattern and immunoprofile staining that overlapped with known types of RCC, but with younger age at presentation and an aggressive clinical course. It highlights the need for in-depth molecular testing in patients with similar presentations, and supports the increasing importance of genetic mutations in classification of renal cancers. NRG1 fusions are potential targets for direct inhibition, and further research is warranted to develop such treatments. However, in light of the rarity of NRG1 fusion RCC, aggregating the cases into a database, similar to the eNRGy1 Global Multicenter Registry for lung adenocarcinoma (29), will help increase our understanding of their behaviour, response to treatment and ideal treatment regimen.

## Methods

### DNA-based next generation sequencing (NGS)

Genomic DNS was extracted from 5X 10μM FFPE sections (Maxwell Promega). Following NGS analysis using a bespoke capture panel (RMH200), no pathogenic variants were identified in the VHL, FH, SDHA, SDHB, SDHC, SDHD, TCEB-1, TSC1, TFE3, TSC2, MET, MTOR and BRAF genes.

DNA samples were sequenced using a targeted, custom-designed capture panel (RMH200Solid panel) consisting of 233 cancer related genes. The panel was validated for detecting single-nucleotide variants (SNVs), small insertions and deletions (indels), copy number variations (CNVs) and selected structural variants (SVs). NGS libraries were prepared from 25-800ng DNA using the KAPA HyperPlus Kit (Kapa Biosystems, Wilmington, MA, USA) and IDT UDI 8bp adapters (Integrated DNA Technologies, Coralville, USA), following the manufacturer’s protocol, including dual-SPRI size selection of the libraries (300-500 bp). To optimise enrichment and reduce off-target capture, pooled, multiplexed, amplified pre-capture libraries (up to 20 samples per hybridization) were hybridized overnight using 1.5 µg of total DNA to a custom design of DNA baits complementary to the genomic regions of interest (HyperCap, Roche Sequencing, Basel, Switzerland). Hybridised DNA was PCR amplified and products purified using AMPure XP beads (Beckman Coulter, Danvers, MA, USA) and quantified using the Qubit dsDNA High Sensitivity Assay Kit with the Qubit 3.0 fluorometer (Invitrogen, Carlsbad, CA), and High Sensitivity D1000 TapeStation (Agilent, Santa Clara, USA).

Sequencing was performed on a NovaSeq6000 (Illumina, San Diego, CA, USA) with 100bp or 150bp paired-end reads and v1 chemistry, according to the manufacturer’s instructions.

The FFPE tissue panel sequencing data were analyzed using an in-house DNA panel pipeline (MDIMSv4). For demultiplexing, bcl2fastq (v2.20.0) was used to isolate reads for each sample. The reads were aligned to the reference genome build GRCh37/Hg19 using Burrows–Wheeler Aligner (BWA-MEM), followed by the marking of PCR duplicates and calculation of various QC metrics using Picard. Genom Analysis ToolKit (GATK) was used for realigning around indels to improve indel calling and base quality score recalibration for adjusting systematic errors made by the sequencer when estimating quality scores of each base call. GATK-Mutect2 was used for variant calling for tumour only analysis and tumour-normal paired analysis. The copy number caller is an inhouse developed pipeline developed in R that utilises Picard tools to generate coverage statistics from NGS sequencing data. These are then compared to a reference file consisting of multiple samples previously sequenced using the same panel in order to remove coverage bias and aid normalisation to generate log2 ratios that are plotted. Segmentation is performed using the R package DNAcopy v1.64.0. A log2 ratio >1 is considered an amplification while <0.5 is a suspected deletion. Finally, Manta (v1.2.2) and Pindel (v0.2.5b8) were used for the detection of structural variants.

All potential mutations, structural variants, and CNVs were visualized using Integrative Genomics Viewer; the following filtering criteria were applied: minimum variant allele depth 10x, Variant Allele Frequency (VAF) threshold at 5%. SNVs can be detected with >98% sensitivity (95% CI) and >96% specificity (95% CI). Small indels detected with sensitivity >89% (95% CI) and accuracy >73% (95% CI) at >5% VAF. This panel is capable of detecting gene amplifications (CNV ratio >2) and deletions (CNV ratio <0.5). Structural variant analysis has been validated for large indels, tandem duplications (BCOR, FLT3, KMT2A) and translocations with sensitivity >98% (>5 reads).

### RNA Sequencing

RNA was isolated from formalin-fixed paraffin embedded (FFPE) tissue sections, using RSC RNA FFPE Kit and Maxwell^®^ RSC 48 Instrument (Promega, WI, USA). Quantifying the RNA concentration with the Qubit 3 Fluorometer (Thermo Fisher Scientific, Waltham MA USA). The TapeStation 4200 (Agilent, Santa Clara, CA, USA) was used for the DV200 determination to classify degraded RNA by size (>70%). Molecular analysis was performed using the TruSight RNA Fusion Panel (Illumina, San Diego, CA, USA.) which comprehensively detects gene fusions and gene expression changes with a focus on 1,385 genes cited in public databases and implicated in cancer. According to the manufacturer’s protocol, 20 ng RNA was used as input. Libraries were paired end sequenced (2x75bp) on a NextSeq 500 (Illumina, San Diego, CA, USA) with 7.3 million reads and a fold coverage of >200x (coding regions). The RNA-Seq alignment app v2.02 (Illumina, San Diego, CA, USA) was employed using the Manta-Fusion algorithm to call fusions. In addition, the Integrative Genomics Viewer (IGV), version 2.12.2 (Broad Institute, San Diego, USA) software was used for data visualisation of the fusion (see **Figure 2**).

## Data availability statement

The original contributions presented in the study are included in the article/supplementary material. Further inquiries can be directed to the corresponding author.

## Ethics statement

The studies involving human participants were reviewed and approved by West of Scotland Research Ethics Committee. The patients/participants provided their written informed consent to participate in this study. Written informed consent was obtained from the individual(s) for the publication of any potentially identifiable images or data included in this article.

## Author contributions

AD and SS did the main writing and editing of the article. SS took the histological images and creation of figures. SM, M-AD, MT, AB, EB were involved in proof-reading and editing contributions of the article draft. All authors contributed to the article and approved the submitted version.
